# Low-voltage scanning electron microscopy study of lampbrush chromosomes and nuclear bodies in avian and amphibian oocytes

**DOI:** 10.1038/srep36878

**Published:** 2016-11-18

**Authors:** Tatiana Kulikova, Tatiana Khodyuchenko, Yuri Petrov, Alla Krasikova

**Affiliations:** 1 Saint-Petersburg State University, Saint-Petersburg, Russia

## Abstract

Nucleus is a highly compartmentalized part of the cell where the key processes of genome functionality are realized through the formation of non-membranous nuclear domains. Physically nuclear domains appear as liquid droplets with different viscosity stably maintained throughout the interphase or during the long diplotene stage of meiosis. Since nuclear body surface represents boundary between two liquid phases, the ultrastructural surface topography of nuclear domains is of an outstanding interest. The aim of this study was to examine ultrathin surface topography of the amphibian and avian oocyte nuclear structures such as lampbrush chromosomes, nucleoli, histone-locus bodies, Cajal body-like bodies, and the interchromatin granule clusters via low-voltage scanning electron microscopy. Our results demonstrate that nuclear bodies with similar molecular composition may differ dramatically in the surface topography and vice versa, nuclear bodies that do not share common molecular components may possess similar topographical characteristics. We also have analyzed surface distribution of particular nuclear antigens (double stranded DNA, coilin and splicing snRNA) using indirect immunogold labeling with subsequent secondary electron detection of gold nanoparticles. We suggest that ultrastructural surface morphology reflects functional status of a nuclear body.

Nuclear structures are complex and dynamic parts of the cell that lack a surrounding membrane^1^,^2^. According to modern concept, nuclear non-membranous domains present as liquid droplets in the nucleoplasm with increased concentration of appropriate proteins and RNAs^3^,^4^. However, these domains possess distinct boundaries separating them from the surroundings and preserving their integrity. Although the inner ultrastructural morphology and molecular composition of main conservative nuclear domains are well characterized, much less is known about ultrastructural topography of their surface. The main reason for that are the difficulties of their isolation from the interphase nuclei. There are only a handful of works describing the ultrathin surface organization of the nuclear bodies in the interphase cells, namely biochemically isolated nucleolus^5^,^6^ and Cajal bodies (CB)^7^. Surface topography of chromatin is quite well-characterized for the mammalian and plant metaphase chromosomes^8^,^9^,^10^ and for the polythene chromosomes from insect salivary glands^11^,^12^. Recent evidence suggest liquid-like structure not only for nuclear domains but also for chromatin^13^.

Giant nuclei of growing avian and amphibian oocytes represent a useful system for exploration of the surface ultrastructure of a number of nuclear bodies as well as transcriptionally active chromosomes. The main advantage here is the large size of the nucleus which allows manual isolation of intact nuclear structures into distinct preparations. Growing oocyte nucleus houses actively transcribed chromosomes in the lampbrush form and, in the case of amphibians, diverse extrachromosomal and chromosome associated nuclear bodies (numerous amplified nucleoli, histone locus bodies (HLB), interchromatin granule clusters (IGC) or B-snurposomes and other nuclear organelles)^14^,^15^,^16^,^17^,^18^. Recently, amplified nucleoli from Xenopus oocyte became a model object in defining the liquid-like behavior of nuclear bodies^19^. In contrast, the avian late-stage oocyte nucleus lacks nucleoli^20^,^21^,^22^ but contains chromosome-attached centromere protein bodies^21^,^23^,^24^. In some species of birds, Cajal body-like (CB-like) bodies were described in the growing oocyte nucleus^23^.

The inner ultrathin organization of the amphibian and avian oocyte nuclear structures has been investigated in depth using transmission electron microscopy (TEM)^23^,^25^,^26^,^27^,^28^,^29^,^30^,^31^. Preparations of the oocyte nuclear content have been used for conventional SEM as well. However, the ultrastructural surface topography was thoroughly described only for the lampbrush chromosomes^29^,^32^,^33^,^34^. There are only few examples of the surface topography of nuclear bodies from amphibian oocytes studied using SEM^32^,^35^, and there is no published data on the surface topography of nuclear bodies from the avian oocytes. Finally, there are no works analyzing the correlations between ultrathin topography and the distribution patterns of marker antigens on the surface of the oocyte nuclear structures.

Low-voltage scanning electron microscopy (LV–SEM) is one of the techniques for analyzing surface topology of various biological samples with high resolution. Compared to conventional SEM, LV–SEM with the secondary electron detection allows for analysis of the surface topography of uncoated, non-osmicated biological samples. Moreover, only in case of LV–SEM one has the opportunity to apply immunogold labelling techniques^36^,^37^.

In this work we have aimed to visualize with high resolution the surface of microsurgically isolated nuclear bodies and giant lampbrush chromosomes from avian and amphibian oocytes. Our results demonstrate that LV–SEM without osmium fixation and conductive coating allows for the identification of extrachromosomal and chromosome-associated nuclear bodies in the oocyte nuclear content preparations. Moreover, individual lampbrush chromosome regions such as chromomeres, lateral loops with specific morphology of RNP-matrix, transcribed and untranscribed looped-out DNA regions are easily recognizable. Although usage of hypotonic treatments, chemical fixation, dehydration, and air-drying for samples preparation disturb liquid properties of nuclear structures, surface ultrastructure is adequately preserved and generally reflects the state of liquid-liquid phase border. Ultrastructural topography of amplified nucleoli, HLB and IGC from amphibian oocytes has been characterized for the first time. We have adapted standard immunogold labelling technique with subsequent secondary electron detection of gold nanoparticles to reveal superficial distribution of certain nuclear antigens namely coilin, small nuclear RNAs (snRNA) and double-stranded DNA (dsDNA) on the oocyte nuclear structures. Based on the results obtained we argue that in spite of similar molecular compositions by surface ultrastructure the oocyte nuclear bodies may differ significantly, although similar surface topological characteristics can be exhibited by structures with different molecular composition. We discuss how the surface morphology may reflect functional status of a nuclear body.

## Material and Methods

### Objects

Oocytes of the domestic chicken (*Gallus gallus domesticus*), Japanese quail (*Coturnix coturnix japonica*), rock pigeon (*Columba livia*) and African clawed frog (*Xenopus laevis*) were used in this study. All animal procedures were conducted in accordance with the Guide for the Care and Use of Laboratory Animals^38^. Fragments of the frog ovary were obtained surgically from animals anesthetized with 0.15% solution of MS-222 (tricaine methanesulfonate) (Sigma). All animal experiments were approved by the Local Animal Ethic Committee of Saint-Petersburg State University (## 131-03-2 and 131-03-3).

### Preparations of the oocyte nuclear content

Preparations of the nuclear content were made according to standard protocols with minor changes^29^,^39^,^40^,^41^,^42^. For oocyte nuclei content preparations standard microscope slides (Menzel) were used. Prior to work slides were washed in 2% 7X cleaning solution (MP Biomedicals), thoroughly rinsed in distilled water and dried at 140 °C in the drying cabinet. All isolation media and solutions were made with deionized water and cell culture grade reagents (Sigma).

Fragment of frog ovary was dissected, placed in OR2 medium (82.5 mM NaCl, 2.5 mM KCl, 1 mM MgCl_2_, 1 mM CaCl_2_, 1 mM Na_2_HPO_4_, 5 mM HEPES; pH 7.8) and incubated at +18 °C overnight. Avian ovaries were dissected, placed in a humidity chamber at +15 °C and used for preparation of nuclear spreads within the next two hours. Both avian and frog oocyte nuclei were isolated one by one using precision forceps or tungsten needles in an embryo dish with 5:1 medium (83 mM KCl, 17 mM NaCl, 6.5 mM Na_2_HPO_4_, 3.5 mM KH_2_PO_4_, 1 mM MgCl_2_, 1 mM dithiothreitol; pH 7.0). Isolated avian oocyte nuclei were immediately washed from the remaining yolk in a hypotonic 1/4 solution (20,7 mM KCl, 4,3 mM NaCl, 1,6 mM Na_2_HPO_4_, 0,9 mM KH_2_PO_4_, 1,0 mM MgCl_2_, 1,0 mM dithiothreitol, 0,1% formaldehyde) and transferred to individual isolation chambers filled with 1:4 solution. To release nuclear content the nuclear envelopes were broken using thin tungsten needles inside isolation chamber. Isolated frog nuclei were left in the 5:1 solution for approximately 10 seconds and then transferred to the embryo dish filled with 1:4 solution, where nuclear envelopes were separated from the nuclear content using thin tungsten needles. All manipulations with the oocyte nuclei were performed under stereomicroscope (Leica MZ9, MZ12 and MZ16); nuclei and nuclear content were transferred individually using standard micropipettes. To disperse nuclear content, preparations were left on ice or at +4 °C for 20 minutes and then centrifuged at 3500 g at +4 °C for 30 minutes.

### Analysis of surface topography

#### Fixation and pretreatments

After the centrifugation step preparations of nuclear content destined for topography analysis with low voltage scanning electron microscopy were fixed in the mixture of 2.5% glutaraldehyde and 2% formaldehyde in 1 × PBS (1.47 mМ KH_2_PO_4_, 4.29 mM Na_2_HPO_4_, 137 mM NaCl, 2.68 mM KCl, pH 7.5) for 30 minutes at room temperature, then dehydrated in a series of ethanol-water solutions of increasing concentration (50%, 70% and 96% ethanol) and dried in air. A single drying technique was used for all preparations. Isolation chambers were removed from the slides during transition from 70% to 96% ethanol. Preparations were analyzed in the wide-field microscope–Leica DMR4000B; selected preparations were imaged at low magnification for easier orientation under LV–SEM.

#### Low voltage scanning electron microscopy

Preparations without any conductive coating and critical point drying were analyzed in scanning electron microscope Zeiss Merlin at low voltage (0.1–0.4 kV) regime. Detector of secondary electrons (In-lens SE) was used for viewing and imaging the surface topography. Following settings were used: working distance −0.4–0.6 mm, chamber pressure in the 10^−6^ Torr range, beam current −90–130 pA. Acceleration voltage in the range of 0.1–0.2 kV was used to investigate the surface topography. The low electron energy and low beam current work to limit radiation damage of the sample investigated. At the same time, lower values of the electron energy correspond to the lower crossover point of the total electron yield^43^, allowing to image an uncoated dielectric specimen without additional charge compensation.

### Analysis of surface antigens distribution

#### Antibodies used

The following primary antibodies were used: mouse monoclonal antibodies (mAb) against double-stranded DNA (dsDNA) (HYB331-01) (Abcam), mAb against 2,2,7-trimethylguanosine cap of snRNA (K121) (Santa-Cruz Biotechnology); rabbit polyclonal serum H-300 against coilin (Santa Cruz Biotechnology). Corresponding secondary antibodies were used: goat anti-mouse IgG (H + L) conjugated with 18 nm colloidal gold nanoparticles (Jackson Immunoresearch Lab); goat anti-rabbit IgG conjugated with 10 nm colloidal gold nanoparticles (Sigma).

#### Fixation

For immunogold labeling, preparations of the oocyte nuclear content were made as described elsewhere^42^. Slides were fixed in 2% formaldehyde in PBS (pH 7.5) for 30 minutes at room temperature and then dehydrated in 70% ethanol. Prior to being removed from the slides, isolation chambers were filled with 70% ethanol and covered with cover slips to facilitate imaging of the wet preparations in the wide-filed microscope for analysis and selection purposes.

#### Immunogold labeling

Nuclear content preparations selected for immunogold labeling were rehydrated in a series of ethanol-water solution of decreasing concentration (70%, 50%, 35%) and then transferred to PBS for 10 minutes. Rehydrated preparations were then incubated with blocking solution (0.5% blocking reagent (Millipore) in PBS), followed by primary and secondary antibodies, both diluted in blocking solution. All incubations were made in a humidity chamber at room temperature, for one hour each. After every antibody incubation step, preparations were washed three times with PBS at room temperature. For double immunolabeling, two antigens were detected sequentially. Upon completion of the first antigen immunolabeling, preparations were fixed in 2% formaldehyde in PBS for 15 minutes, washed in three changes of PBS, and then the second antigen was immunolabelled. Antigens detected with larger (18 nm gold) conjugates were immunolabeled first. After immunogold labeling the preparations were fixed in a 2.5% glutaraldehyde solution in PBS (pH 7.5) for 15 minutes, dehydrated in a series of ethanol-water solution of increasing concentration (50%, 70% and 96%) and air dried.

### Low voltage scanning electron microscopy

Immunogold labeled preparations were analyzed using Zeiss Merlin scanning electron microscope in a low voltage regime. Acceleration voltage of 0.4 kV was used for the imaging of gold nanoparticles; other parameters were the same as for the surface topography analysis. In the case of 0.4 kV acceleration voltage, the voltage signal of secondary electrons contains more SE2 electrons excited by back-scatter compared to the of secondary electron voltage signal with the 0.2 kV acceleration voltage^43^. These SE2 electrons produce material contrast used to distinguish gold nanoparticles when using detector of secondary electrons (In-lens SE) for viewing and imaging sample topography. In-column detection of back-scattered electrons (EsB) was used to validate presence of colloidal gold particles.

### Image acquisition and measurements

Two modes of the image acquisition were used: scanning of a single frame with a high pixel dwell time, or scanning of a series of 10 frames with subsequent averaging. The modes were changed to overcome the charging of the surface of the sample. The pixel size varied from 3 nm to 150 nm for different magnifications, and a pixel dwell time was in a range of tens of microseconds (up to 100 μs) for a single scan or around 10 μs for scanning with subsequent averaging. Total acquisition time was in a range of 1–5 minutes. For all images electron dose was of about 2–4 × 10^4^ electrons per pixel, or from 10^14^ to 10^17^ electrons per cm^2^ depending on magnification. No posterior filtering or noise reduction was used. The radiation damage rate was quite low due to low energy of the electron beam, so it was not observed after ten scans at high magnification; whereas electron beam induced hydrocarbon contamination appears after the second image acquisition. Measurements of ultrastructural elements were performed using tools of SmartTiff (Ziess) software; at least five examples of the same ultrastructural element were measured. For more explicit presentation of nanoparticle distribution layer masks were created in Adobe Photoshop. The mask is a separate image which mirrors contours of a nuclear structure and distribution of gold nanoparticles.

## Results and Discussion

Previous high-resolution SEM images of loops with specific morphology on avian and amphibian lampbrush chromosomes were obtained after gold or palladium coating and osmium fixation^29^,^33^. In this study we have applied low-voltage scanning electron microscopy (LV–SEM) to analyze the surface topography of the nuclear structures from avian and amphibian oocytes. For LV–SEM surface topography analysis of different nuclear structures we have adopted 2% formaldehyde and 2.5% glutaraldehyde fixation followed by ethanol dehydration.

### Lampbrush chromosome morphology

The surface of the following lampbrush chromosome regions was examined with LV-SEM: chromomeres, interchromomeric regions, transcriptionally active simple lateral loops, certain complex loops and chromosome associated structures.

#### Axes and chromomeres

**Figure 1:**
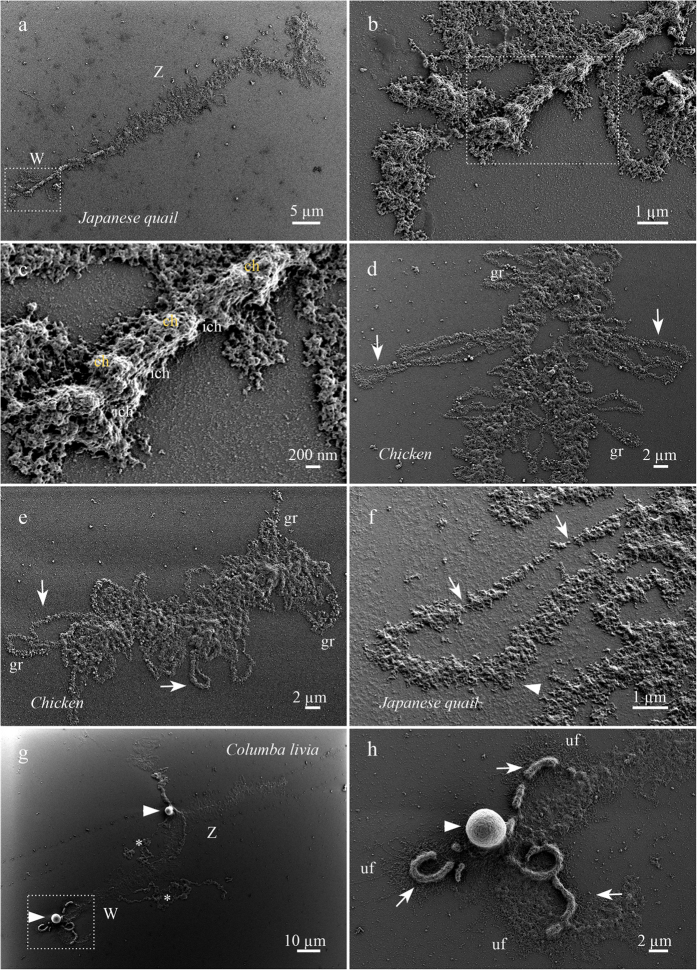
The surface morphology of avian lampbrush chromosomes: chromomeres, lateral loops, and associated structures. Lampbrush chromosomes of *Coturnix coturnix japonica* (**a–c,f**), *Gallus gallus domesticus* (**d,e**) and *Columba livia* (**g,h**). (**a–c**) Surface morphology of chromomeres in the ZW-lampbrush bivalent. (**c**) Enlarged fragment of W-lampbrush chromosome demonstrates compact neighboring chromomeres (chr) and interchromomeric regions (ich). (**d,e**) Fragment of macrobivalent and whole microbivalent demonstrate simple lateral loop morphology (arrows); gr–large 100–200 nm RNP-granules. (**f**) Enlarged fragment of simple lateral loop; RNP-matrix of a transcription unit (arrowhead), short regions of “nacked” loop axis (arrows). (**g,h**) The morphology of ZW-bivalent with pericentromeric dense loops, centromere protein bodies (arrowheads) and giant terminal RNP-aggregates (GITERA) (asterisks). (**h**) Enlarged fragment of panel (**g**), W-chromosome with centromere protein body (arrowhead) and pericentromere dense loops (arrows); uf–untwisted RNP-fibrils. Low-voltage scanning electron microscopy. Scale bars: (**a**) 5 μm, (**b**,**f**) 1 μm, (**c**) 200 nm, (**d**,**e**) 2 μm, (**g**) 10 μm, (**h**) 2 μm.

Axes of lampbrush chromosomes consist of the chromomeres connected by relatively short interchromomeric regions linking individual chromomeres. The morphology of chromosome axis was analyzed on an example of a compact W-chromosome which carries less lateral loops (Fig. 1a–c). On the majority of the others avian lampbrush chromosomes axial structures appears to be hidden by numerous small lateral loops covering chromosome axis (Fig. 1d,e). For this reason lampbrush chromomere surface had not been described in earlier conventional SEM study^32^. As can be seen in the high magnification LV-SEM images, the surface of both chromomeres and interchromomeric chromosome axes consists of fibrils approximately 30 nm in diameter (Fig. 1c). In the chromomeric regions the surface of the chromosome axes looks like a dense tangle of these 30-nm fibrils. The dimensions of the fibrils correspond to the size of the “unraveled skeins” of deoxyribonucleoproteins at the periphery of the chromomeres described on ultrathin sections of nuclear content preparations of amphibian oocytes analyzed by TEM^25^. In our preparations the individual chromatids in the interchromomeric regions were indistinguishable and neighboring chromomeres appeared to be linked by wide longitudinal elements consisting of the same 30-nm fibrils (Fig. 1b,c). Formation of such longitudinal elements can be explained by chromatin compaction in the chromomeres with participation of proteins of the condensin complex^18^. Earlier interchromomeric regions had been visualized in TEM study of negatively stained and mechanically stretched lampbrush chromosomes of newt^44^ as 20 nm fibers composed by two sister chromatids. 30-nm fiber has been thought to be the next to nucleosome level of chromatin organization and was described in SEM studies of mitotic^45^ and meiotic chromosomes^46^. However recent data disprove the existence of 30 nm chromatin fiber *in vivo*^47^. Formation of 30 nm fibers in isolated chromosomes was found to be a consequence of hypotonic solution treatment during samples preparation^48^.

#### Centromeres

The organization of centromere regions in the lampbrush chromosomes was described in detail in our recent review^49^. Centromere regions of chicken, Japanese quail and clawed frog lampbrush chromosomes are associated with small centromere granules^50^, whereas centromeres of pigeon are marked by centromere protein bodies (Fig. 1g,h). Interestingly, on LV-SEM images the centromere regions of chicken, Japanese quail and clawed frog lampbrush chromosomes were morphologically indistinguishable. Such homogeneity of the lampbrush chromosome axes in the centromere region and chromosome arms can be seen on an example of chicken microchromosome (Fig. 1e) and W lampbrush chromosomes of Japanese quail (Fig. 1b,c). Сentromere granules are not visible in LV-SEM images alternatively since their ultrastructural morphology is similar to chromatin of chromomeres or they are embedded into surrounding chromatin.

#### Lateral loops

Lampbrush chromosomes carry lateral loops of two major morphological types: one, the prevailing type of lateral loops, is called simple; another type is rare, loci-specific loops called complex loops or loops with complex morphology^16^,^18^. Simple lateral loops bear one or several transcription units, the direction of transcription in which is clear due to the thickening of the RNP-matrix consisting of nascent RNPs (Fig. 1d–f). Thickness of the RNP-matrix on simple lateral loops does not correspond to the length of transcriptional unit due to co-transcriptional folding, packaging and splicing of the nascent transcripts^51^.

Individual transcription units are separated from each other by short regions of “naked” loop axis 10 to 20 nm thick (Fig. 1f). The RNP-matrix consists of compactly packed globules 40 to 50 nm in diameter (Supplementary Fig. 1). With increase of the RNP-matrix thickness, the compact large granules of 100–200 nm may appear on the surface of the RNP-matrix closer to the ends of long transcription units (Fig. 1d,e). In the earlier works the size of basic RNP-particles comprising the RNP-matrix of simple lateral loops varied from 20 nm^52^, to 26 nm^53^ or to 30 nm^28^,^32^ depending on the EM technique applied. Interestingly, in polytene chromosomes from insect salivary glands studied by conventional SEM, in the highly transcriptionally active loci called Balbiani ring, the size of the majority of nascent RNP-particles varied from 35 to 55 nm^11^,^12^. Further research has demonstrated that the elementary subunit of Balbiani ring granules consists of 10 to 12 RNA-containing particles of 10 nm in diameter^54^. At the same time, nascent transcripts in the interphase nucleus present as perichromatin fibrils of 3–5 nm in diameter^55^,^56^. The size of the nascent RNP-granules observed in our study resembles RNP-granules of Balbiani rings on polytene chromosomes and does not correspond to the perichromatin fibrils of interphase nuclei. This may reflect the next packaging level of the nascent RNPs in the hyperactive and longer transcription units such as lampbrush chromosome lateral loops or Balbiani rings. Another type of the RNP-structures in the interphase nucleus, –the perichromatin granules, –may correspond to RNP-granules observed in our study.

Organization of the RNP-matrix in complex loops differs from that found in simple loops. LV-SEM revealed that the RNP-matrix of dense loops arising from the pericentromere region of pigeon lampbrush chromosome W consists of compactly packed RNP-fibrils forming thick cylindrical structures of 600 to 700 nm in diameter (Fig. 1g,h; Supplementary Fig. 1). Large volume of RNP-matrix may reflect the length of nascent transcripts synthesized in the loci of dense loop formation. Fibrils of the individual transcripts can untwist from the RNP-matrix of dense loops due to centrifugation of the sample, apparently (Fig. 1h; Supplementary Fig. 1). The length of an individual untwisted RNP-fibril may reach 8 μm which corresponds well to the contour length of a dense loop. The thickness of an individual RNP-fibril untwisted from RNP-matrix of the dense loop varied from 30 to 50 nm, thus corresponding to the thickness of the RNP-fibrils comprising RNP-matrix of simple loops. Similarly, morphological variations between different types of complex loops on amphibian lampbrush chromosomes were found to be determined by the spatial organization of the RNP-matrix with universal size of the basic RNP-particle^27^,^57^.

Besides complex loop-based structures, lampbrush karyotypes of many avian species are characterized by formation of giant terminal RNP-aggregates (GITERA)^58^. GITERA are truly giant nuclear structures reaching up to 100 μm in length and 20 μm in diameter, exact size depending on species^59^,^60^ and do not acquire form of polarized RNP-matrix corresponding to a transcription unit (Fig. 1g). LV-SEM revealed that GITERA consist of tightly packed and chaotically arranged RNP-fibrils with the same thickness as the RNP-fibrils of simple lateral loops (Supplementary Fig. 1).

### Nuclear bodies morphology

**Figure 2:**
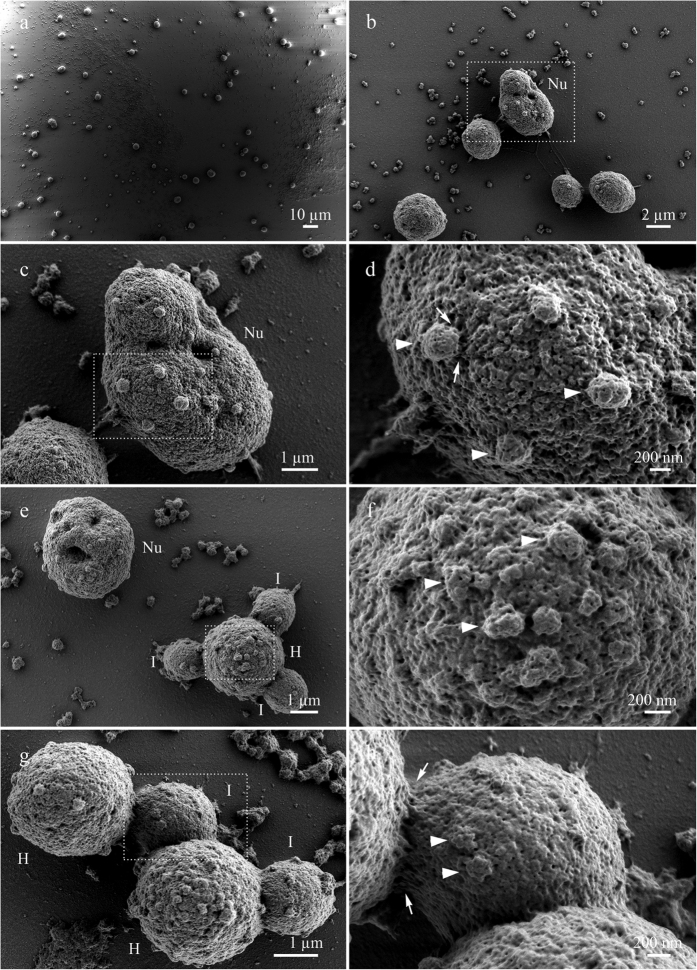
The surface morphology of nuclear bodies from growing oocytes of *Xenopus laevis*. (**a**) Numerous nuclear bodies of *Xenopus laevis* on nuclear content preparations: lampbrush chromosomes, amplified nucleoli, HLB and IGC; (**b–d**) sequential enlarged images demonstrate nucleolus (Nu) with patches (arrowheads) connected to the surface of nucleolus by fibrillar material (arrows); (**e**) – nucleolus (Nu) and HLB (H) with attached IGC (I); (**f**) surface of the enlarged fragment of HLB bearing patches (arrowheads). (**g**) Two HLB (H) with attached IGC (I). (**h**) Enlarged fragment of IGC with the area of contact between HLB and IGC demonstrating fibrillar material (arrows); arrowheads indicates patches. Low-voltage scanning electron microscopy. Scale bars: (**a**) 10 μm, (**b**) 2 μm, (**c**) 1 μm, (**d**) 200 nm, (**e**) 1 μm, (**f**) 200 nm, (**g**) 1 μm, (**h**) 200 nm.

LV-SEM revealed distinctive ultrastructural topography of the oocyte nuclear bodies. Numerous and diverse extrachromosomal bodies (amplified nucleoli, HLB and IGC or B-snurposomes) in the *Xenopus* oocyte nucleus can be seen in Fig. 2(a,b).

#### Nucleolus

Amplified nucleoli of the *Xenopus* oocyte demonstrated high-relief and structured surface with furrows and ridges consisting of granules 40 to 60 nm in size clustered around pores (Fig. 2c,d). Amplified nucleoli have usual tripartite organization with loosely structured peripheral granular component^61^ with the diameter of the granules ranging from 25 to 50 nm^62^. Thus, nucleolar surface corresponds to granular component of the nucleolus representing maturing ribosome particles. According to TEM data, however, the granular component of the nucleolus is composed of particles 15 to 20 nm in size^63^. At the same time, nucleoli isolated by sonication from cultured cells and visualized by SEM have also demonstrated granular surface^5^,^64^, and the size of the granules was estimated at 75–100 nm^5^. Interestingly, the surface of nucleoli on *Chironomus* polytene chromosomes possesses a similar granular nature with size of the granules varying from 16 to 40 nm^12^. It is possible that such differences in nucleolar surface morphology reflect differences in transcriptional activity of ribosomal genes. On the other hand, ultrastructural studies have shown that granular component contains not only ribosomal particles but also possesses considerable protein content^65^.

On the surface of the *Xenopus* oocyte nucleoli we have found rare 200–350 nm-sized patches consisting of less prominent granules and connected to the nucleolus by fibrous material (Fig. 2d).

#### Histone locus bodies

Coilin-containing extrachromosomal nuclear bodies from amphibian oocytes called HLB displayed loosely packed fibrils (30–40 nm in diameter) on the surface rich with cavities and pores (Fig. 2f). The surface of the HLB also bore patches similar to ones observed in nucleolus. The morphology of the patches was similar, but the HLB’s patches are more variable in size and seem to be more embedded and/or budding off from the surface of the body (Fig. 2f). Fibrils on HLB surface were visually similar to the coiled rod-like structures observed in field emission SEM study of CB isolated from the HeLa nuclei^7^. The observed surface ultrastructure is in agreement with the data on high permeability of Xenopus oocyte HLB^66^. TEM of coilin-containing extrachromosomal bodies from amphibian oocytes revealed 30–50 nm sized granules in *Notophtalmus*^14^, 20 nm thick RNP-containing fibrils in *P. waltl*^28^, and two types of granules: small 25 nm and larger 30–40 nm in the *Xenopus* oocytes^17^. Differences in the size of the fibrils of coilin-containing structures can be explained by varying degree of coilin aggregation.

#### Interchromatin granule clusters

HLB can possess from zero to dozens of spherical structures called B-snurposomes–analogues of IGC in *Xenopus* oocytes^17^. LV-SEM revealed that IGC are attached to HLB by fibrillar material (12–25 nm thick fibrils) (Fig. 2g,h). The surface of the IGC is porous and grainy (Fig. 2h) indicating that they consist of granules 30–40 nm in size. TEM studies of the amphibian (*Xenopus* and newt) oocyte revealed that IGC are composed from 20–30 nm sized granules^14^,^30^ similar to the average diameter of the particles making up IGC of the interphase nucleus^55^. B-snurposomes bore rare 100 nm-sized flat granular patches similar to those observed in the HLB. Earlier TEM studies revealed 100 nm patches comprised of 10 nm granules on the surface of the *Pleurodeles* B-snurposomes^16^,^67^. We suggest that the patches observed in our study correspond to the 100 nm patches shown previously in the newt oocytes.

#### Cajal body-like bodies from pigeon oocytes

**Figure 3:**
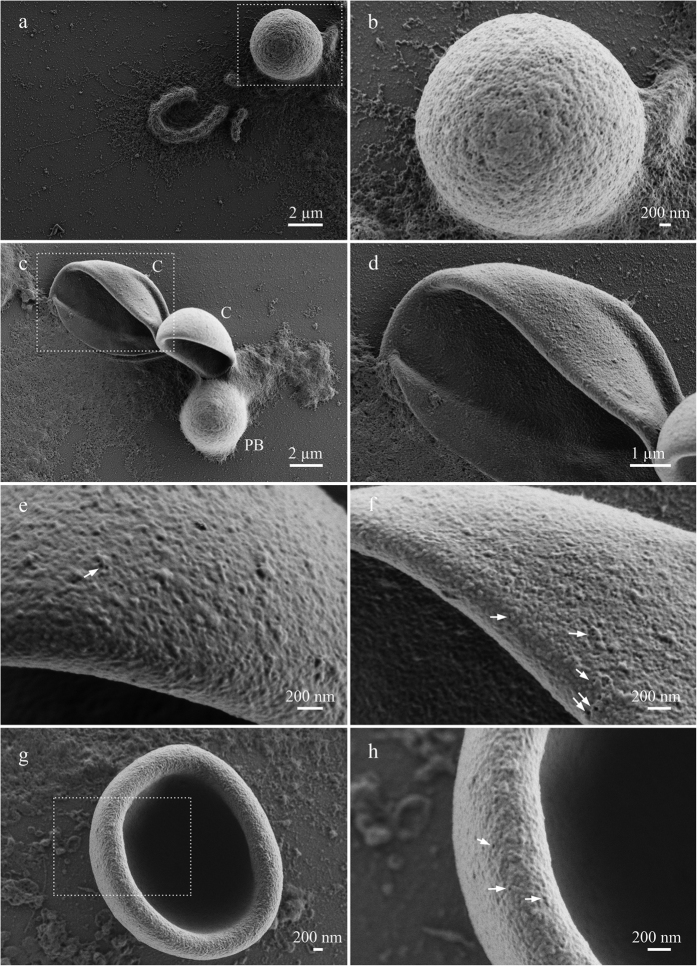
The surface morphology of nuclear bodies from growing oocytes of *Columba livia*. (**a,b**) centromere protein body; (**c**) CB-like bodies (C) and centromere protein body (PB); (**d,g**)–CB-like body; (**e,f,h**) enlarged fragments of CB-like bodies shown on panels (**c,g**); single or grouped pores–arrowheads. Low-voltage scanning electron microscopy. Scale bars: (**a**) 2 μm, (**b**) 200 nm, (**c**) 2 μm, (**d**) 1 μm, (**e–h**) 200 nm.

Coilin-containing spherical bodies in the avian and amphibian oocytes demonstrated quite different topography (Fig. 2e–g; Fig. 3c–h). Extrachromosomal CB-like bodies isolated from pigeon oocyte nucleus demonstrated solid and tuberous surface with flat tubercles of about 30 nm in diameter and rarely scattered single or grouped pores from 20 to 50 nm in diameter (Fig. 3e–h). The surface of CB-like bodies appeared to be the smoothest and least textured of all examined nuclear body surfaces. The smooth surface ultrastructure reflects compact and fibrillar nature of the CB-like body matrix. Earlier TEM observations of nuclear bodies within pigeon oocyte nucleus have shown that the CB-like bodies have extremely electron-dense and homogenous ultrastructure with high affinity to heavy metal contrasting agents^23^. Interestingly, TEM did not reveal tubules or galleries in the matrix of CB-like bodies indicating that pores observed by LV-SEM are superficial and dead-ended. By their morphology and morphodynamics CB-like bodies from pigeon oocytes resemble CBs from oocytes of cricket, the *Acheta domesticus*^68^,^69^.

#### Centromere protein bodies

Centromere protein bodies, typical for oocytes of certain avian species, are coilin-free and do not accumulate any RNPs^24^,^31^,^70^. In pigeon oocytes, centromere nuclear bodies have demonstrated granular surface composed of 60–100 nm thick fibrils and dotted with pores of various sizes (from 4 to 30 nm) (Fig. 3a,b). Previous TEM imaging studies have shown that the centromere protein bodies consist of moderately contrast filaments, with the subsurface filamentous mass being penetrated by rarely distributed tubules with more electron-dense edges^31^. The absence of any RNPs distinguishes the molecular composition of the centromere protein bodies from the composition of other oocyte nuclear bodies described previously in this paper. The ultrastructural topography of the centromere protein bodies resembles that of *Xenopus* HLB. However, the surface of the centromere protein bodies is smoother, does not contain patches, with numerous grains on their surface being smaller than those on HLB. At the same time, the surface of the centromere protein bodies dramatically differs from the surface of CB-like bodies from pigeon oocytes.

**Figure 4:**
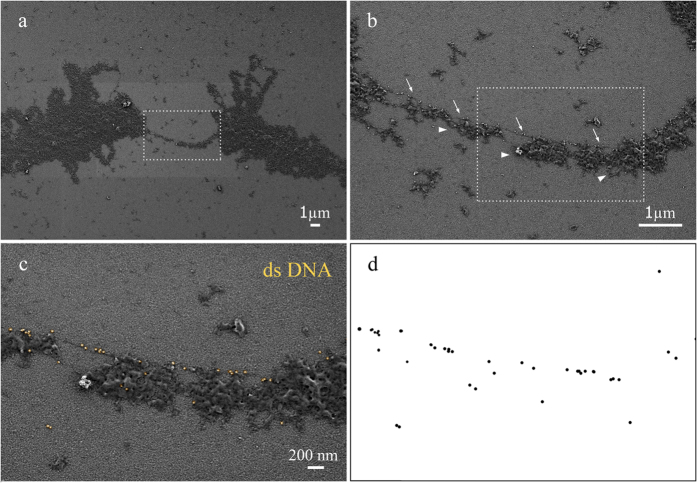
Surface distribution of dsDNA along transcriptionally active regions of lateral loop axes. (**a–c**) Fragment of a lampbrush chromosome of *Coturnix coturnix japonica,* immunogold labelling with antibodies against dsDNA. (**b**) DsDNA revealed by 18 nm gold nanoparticles (electron dense granules) along the DNP-axis (arrows) of individual lateral loop with growing nascent RNP fibrils (arrowheads); (**c)** gold nanoparicles pseudocolured with yellow; (**d**) the distribution pattern of gold nanoparticles.

Summarizing morphological data it should be noted that in our study RNP-particles (nascent RNP on lateral loops, granules on the surface of nucleoli and IGC), in contrast to other measured ultrastructural elements, appeared to be about 10 nm larger than in conventional SEM or TEM studies. We suggest that size increase is caused by hydrocarbon contamination after electron beam damage of uncoated preparations during image acquisition. Moreover, different ultrastructural elements may have different sensitivity to radiation damage, namely RNP-particles are more sensitive than chromatin or coilin fibrils. Indeed, in our recent atomic force microscopy study of similarly prepared lampbrush chromosomes nascent RNP-particles of lateral lampbrush loops were evaluated as 25 nm^71^ which well corresponds to conventional SEM data^32^. Electron beam induced modification can be seen on Supplementary Fig. 2 and on Fig. 4a as a bright rectangle of a previously scanned area.

### Distribution of the double-stranded DNA, coilin and snRNAs on the surface of the oocyte nuclear structures

The immunogold labeling technique was applied to analyze surface distribution of the components typical of nuclear content. We have analyzed the distribution of double-stranded DNA (dsDNA), coilin and snRNAs exposed to the surface of nuclear structures isolated. To this purpose we have adapted the fixation protocol used for oocyte nuclear content preparations with only formaldehyde fixation step followed by ethanol dehydration. The diameter of the entire labeling complex was about 26 nm for 10 nm and 34 nm for 18 nm colloidal gold conjugates correspondingly, which only allows binding to the surface antigens.

The DNA exposed on the surface of the chromatin domains was revealed using immunogold labeling with antibodies against dsDNA (Supplementary Figs 2 and 4) on example of avian lampbrush chromosomes. Tight immunogold labeling pattern of DNA was observed in large compact chromomeres, for instance in the highly compacted W chromosome (Supplementary Fig. 2). At the same time, in less compact chromomeres a less intense labeling pattern was observed with the lateral loops axes also being labeled with antibodies against dsDNA (Supplementary Fig. 2). Untranscribed and thicker regions of lateral loop axes were fully covered by gold nanoparticles (Supplementary Fig. 2). These regions of lateral loops apparently correspond to the regions of lampbrush chromatin enriched with the 5-methylcytosine^72^ (and our unpublished data).

Within transcriptional units of lateral loops the colloidal gold nanoparticles were evenly distributed along regions of “naked” loop axis (Fig. 4b,c). The thickness of the DNP axis was estimated at 10 nm, which corresponds to the nucleosome level of chromatin packaging. We conclude that immunogold labeling followed by LV–SEM reveals dsDNA on the surface of both transcribed and untranscribed looped-out chromatin in avian and amphibian lampbrush chromosomes.

It is known that heterogeneous group of bodies to which CB, HLB, extrachromosomal CB-like bodies and “pearls” belong contain protein coilin and snRNPs (Supplementary Fig. 3)^23^,^73^,^74^,^75^,^76^,^77^. To examine the distribution of marker components on the surface of those bodies we have used antibodies against coilin and antibodies against TMG-cap of the snRNAs as well as corresponding secondary antibodies coupled to 10 nm and 18 nm colloidal gold particles.

Coilin was evenly distributed across the surface of the HLB (Supplementary Fig. 4). The distribution of snRNAs on the surface of the bodies was also regular, devoid of any specific associations (Supplementary Fig. 4). IGC attached to HLB were moderately labeled by gold nanoparticles marking snRNA but not coilin (Supplementary Fig. 4). Numerous fibrillar cords attaching IGC to HLB were not covered by gold nanoparticles marking coilin or snRNA (Supplementary Figs 4 and 5). To simplify the qualitative examination of colloidal gold nanoparticles distribution on the surface of the structures we have approximately evaluated pattern of signal distribution (Supplementary Figs 4 and 5). The pattern clearly supports rather proportional distribution of snRNAs and coilin on the surface of HLB.

On the surface of pigeon CB-like bodies, coilin and snRNAs were also evenly distributed (Fig. 6a,c,e). It is worth noting that centromere protein bodies present in the same image were not labeled with antibodies against coilin (Fig. 6c) as expected from their molecular composition. Interestingly, pigeon CB-like bodies demonstrated denser labeling pattern in regards to coilin if compared to the *Xenopus* HLB. At the same time density of superficial snRNAs were comparable for both nuclear bodies. Higher concentration of coilin may be associated with lower functional activity of CB-like bodies.

**Figure 5:**
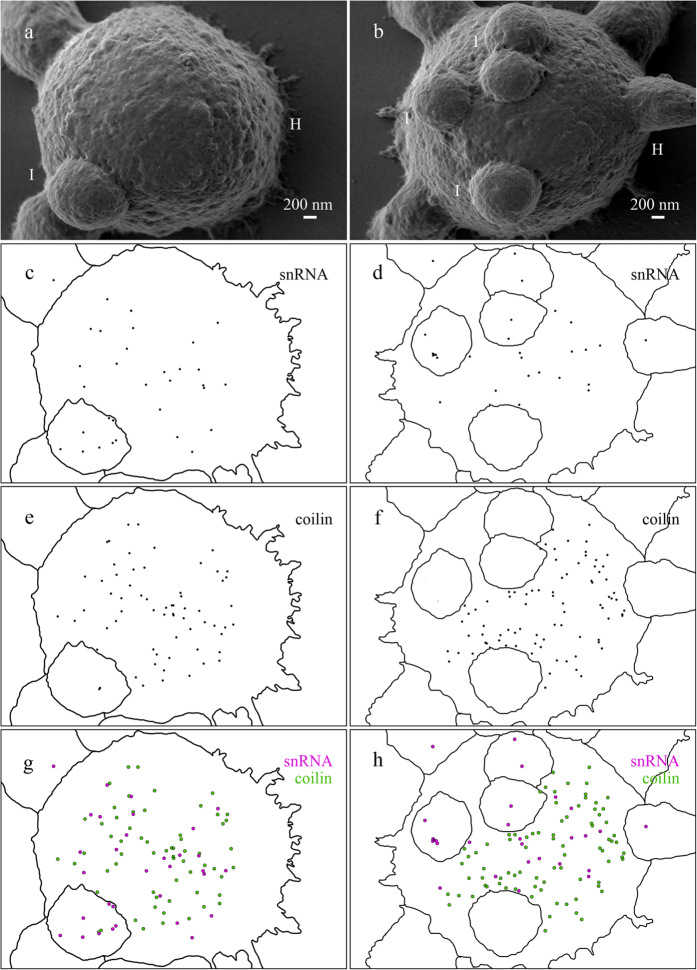
The relative distribution of coilin and snRNA on the surface of HLB and attached IGC of *Xenopus laevis* growing oocyte. Double immunogold labeling with antibodies against coilin and snRNAs (**a,b**). Distribution of coilin and snRNA on the surface of HLB (H) and IGC (I) revealed by 10 nm and 18 nm gold nanoparticles (electron dense granules) correspondingly. (**c,d**) Distribution pattern of gold nanoparticles reflects surface distribution of snRNA. (**e,f**) Distribution patterns of gold nanoparticles reflect surface distribution of coilin; (**g,h**) Distribution pattern gold nanoparticles reflects surface distribution of both coilin (green) and snRNA(magenta). Low-voltage scanning electron microscopy. Scale bars: 200 nm.

**Figure 6:**
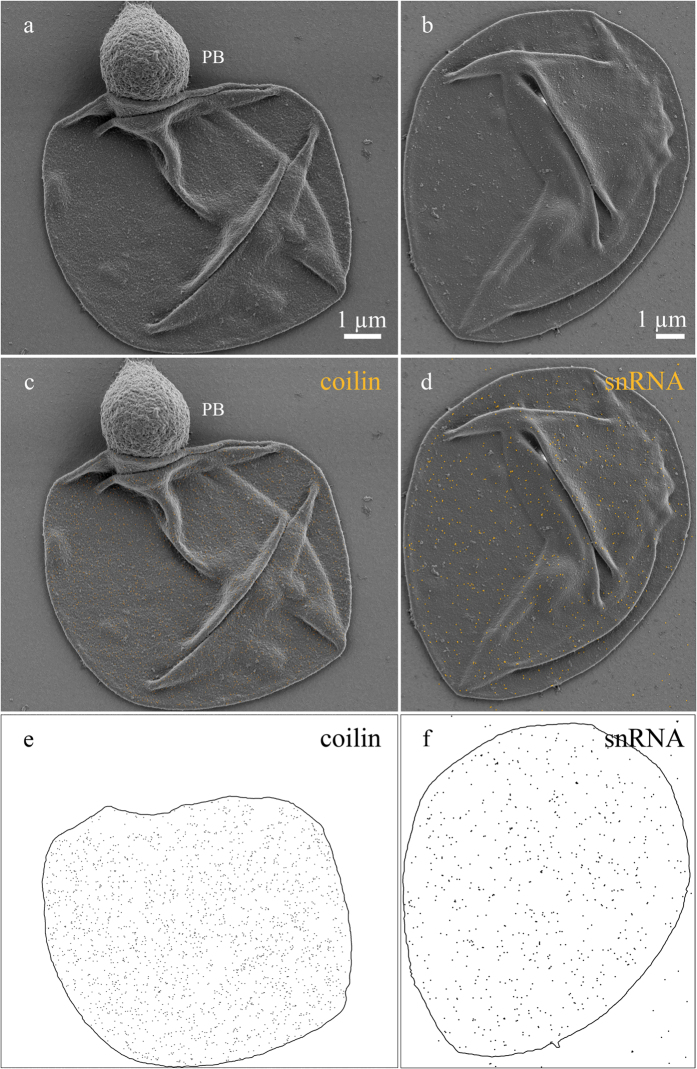
The distribution of coilin and snRNA on the surface of CB-like bodies of *Columba livia* growing oocytes. Immunogold labeling with antibodies against coilin (**a,c,e**) and snRNAs (**b,d,f**); centromere protein body (PB). (**a,b**) Distribution of coilin or snRNA on the surface of CB-like bodies revealed by 10 nm and 18 nm gold nanoparticles (electron dense granules) correspondingly; (**c**,**d**) gold nanoparicles pseudocolured with yellow. (**e**,**f**) Distribution patterns of gold nanoparticles. Low-voltage scanning electron microscopy. Scale bars: (**a**,**b**) 1 µm.

**Figure 7:**
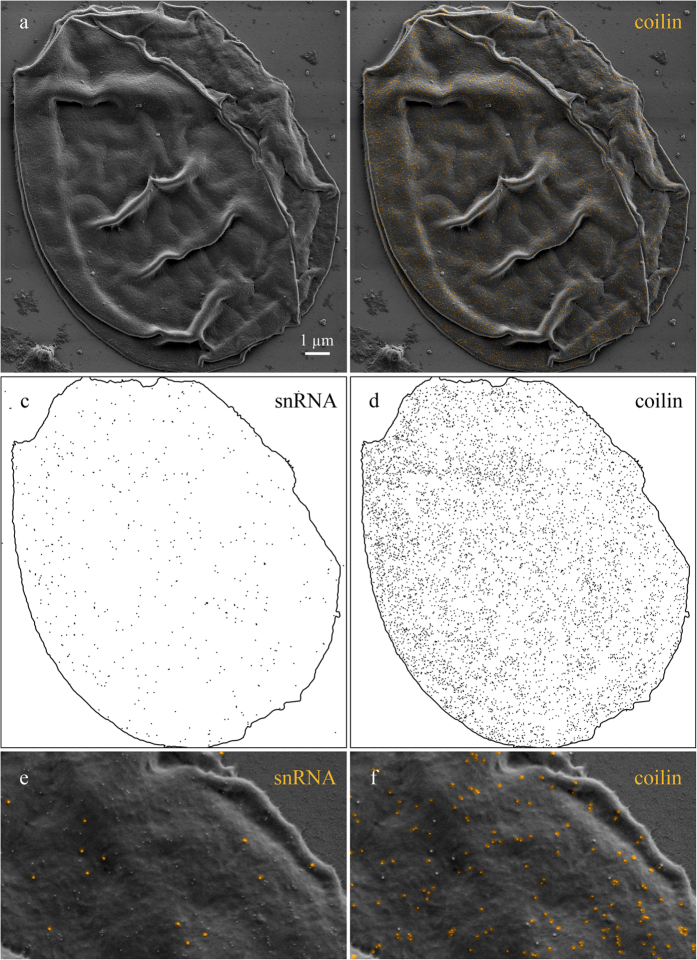
The relative distribution of coilin and snRNA on the surface of CB-like bodies of *Columba livia* growing oocyte. Double immunogold labeling with antibodies against coilin and snRNAs (**a,b**). Distribution of coilin and snRNA on the surface of CB-like body revealed by 10 nm and 18 nm gold nanoparticles (electron dense granules) correspondingly. (**b**) Gold nanoparicles corresponding to coilin pseudocolured with yellow. (**c,d**) Distribution patterns of gold nanoparticles reflect snRNA and coilin surface distribution. (**e,f**) Enlarged images of CB-like body shown on panel (**b**); gold nanoparicles corresponding to coilin and snRNAs pseudocolured with yellow. Low-voltage scanning electron microscopy. Scale bar: 1 μm.

To examine relative localization of coilin and snRNAs we used sequential double immunogold labeling, with 18 nm colloidal gold particles applied first. We have shown that coilin and snRNAs did not co-localize both on *Xenopus* HLB (Fig. 5) and on pigeon CB-like bodies (Fig. 7).

The findings from this study indicate that coilin-rich nuclear bodies in amphibian and avian oocytes demonstrate significant morphological differences in the surface organization on ultrathin scale. At the same time the RNP-containing and proteinaceous nuclear bodies demonstrate similar surface morphology despite significant dissimilarity in their molecular composition. The observations suggest that surface morphology reflects functional status of a nuclear body. Nuclear bodies with a dynamic traffic and biogenesis of the macromolecules probably have complex and loose organization of the surface, while nuclear bodies involved only in RNP storage may possess more solid and homogeneous surface morphology. Also, nuclear bodies involved in storage display higher concentration of structural protein on their surface. At the same time stable morphology of the nuclear bodies likely depends on low-complexity proteins and architectural RNAs^4^. We also show that dsDNA is exposed on the surface of transcriptionally active and inactive chromatin. Finally, based on the results obtained, we conclude that low-voltage scanning electron microscopy without conductive coating is an excellent method that allows examination of intact ultrastructure of oocyte nuclear content and evaluation distribution of the superficial antigens using standard immunogold labeling technique.

## Additional Information

**How to cite this article**: Kulikova, T. *et al*. Low-voltage scanning electron microscopy study of lampbrush chromosomes and nuclear bodies in avian and amphibian oocytes. *Sci. Rep.* **6**, 36878; doi: 10.1038/srep36878 (2016).

**Publisher’s note**: Springer Nature remains neutral with regard to jurisdictional claims in published maps and institutional affiliations.

## Supplementary Material

Supplementary Information
