# Supplementary material for: Low-voltage scanning electron microscopy study of lampbrush chromosomes and nuclear bodies in avian and amphibian oocytes
Source: Sci Rep. 2016 Nov 18;6:36878. doi: 10.1038/srep36878 (PMC5114574; doi:10.1038/srep36878)
Supplement: Supplementary Information [file srep36878-s1.pdf]

Supplementary Information

LOW-VOLTGE SCANNING ELECTRON MICROSCOPY STUDY OF LAMPBRUSH CHROMOSOMES AND NUCLEAR BODIES IN AVIAN AND AMPHIBIAN OOCYTES

Tatiana Kulikova[1], Tatiana Khodyuchenko[1], Yuri Petrov[1], Alla Krasikova[1]*

[1]*Saint-Petersburg State University, Saint-Petersburg, Russia*

*Address for correspondence (Saint-Petersburg State University, 7-9, Universitetskaya nab., 199034, Saint-Petersburg, Russia Tel: 79213267671; E-mail: alla.krasikova@gmail.com)

Supplementary Information Guide

This file includes

Supplementary Figures and Legends S1-S4

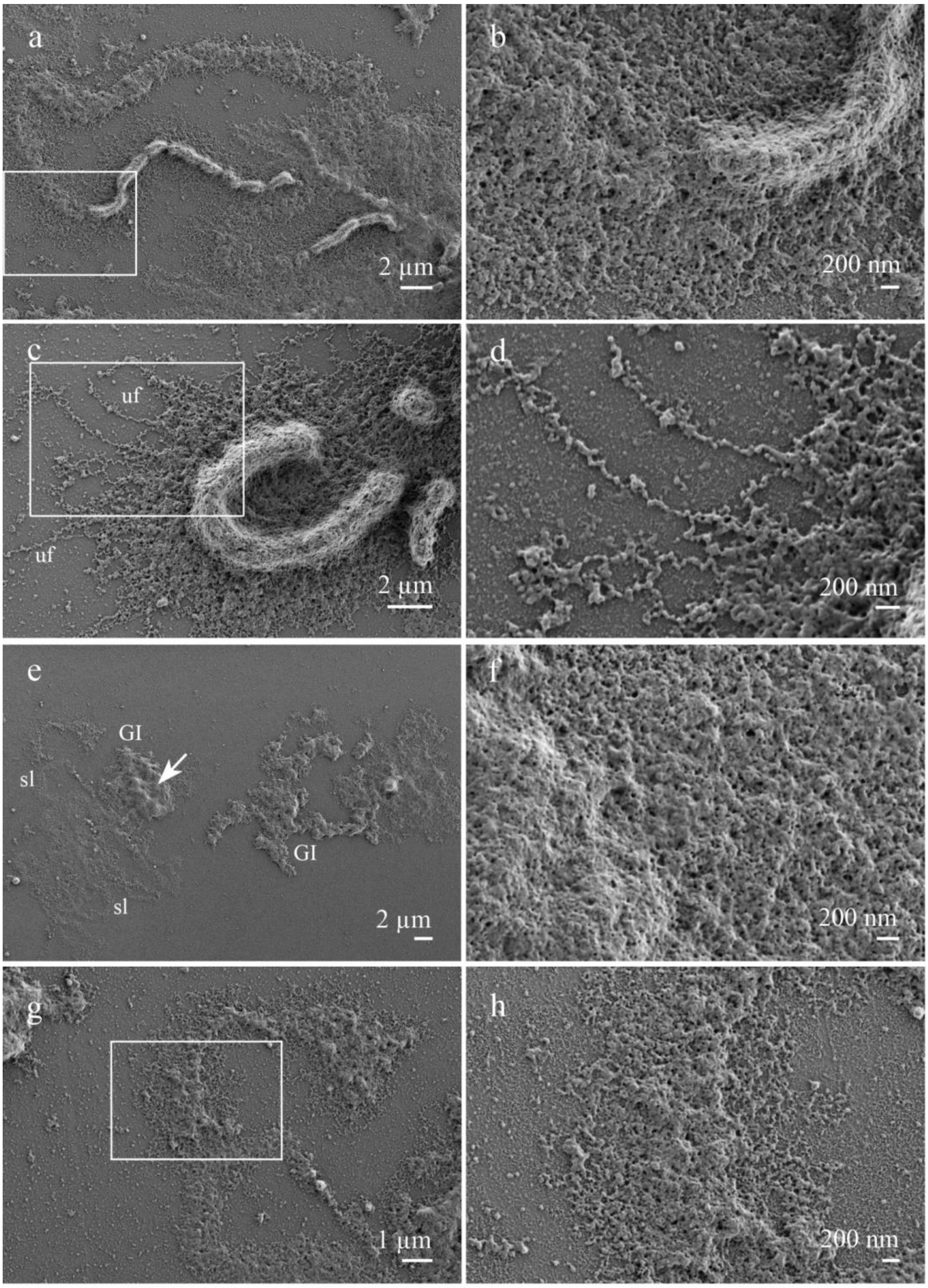
a
2 µm
b
200 nm
c
uf
uf
2 µm
d
200 nm
e
GI
sl
GI
sl
2 µm
f
200 nm
g
1 µm
h
200 nm

Supplementary Figure 1. The surface morphology of complex lateral loops, simple lateral loops and GITERA. Lampbrush chromosomes of *Columba livia*. **a, c** – pericentromere dense loops on chromosome W; **b, d** – enlarged fragments of **a, c**; uf – untwisted RNP-fibrils. **e** – fragment of lampbrush chromosomes bearing GITERA; sl – simple lateral loops, GI – GITERA, arrow – area enlarged on **f**. **f** – enlarged fragment of GITERA. **g, h** – enlarged fragments of simple lateral loops shown on panel **e**. Low-voltage scanning electron microscopy. Scale bars: **a, c, e** – 2 μm, **b, d, f, h** – 200 nm, **g** – 1 μm.

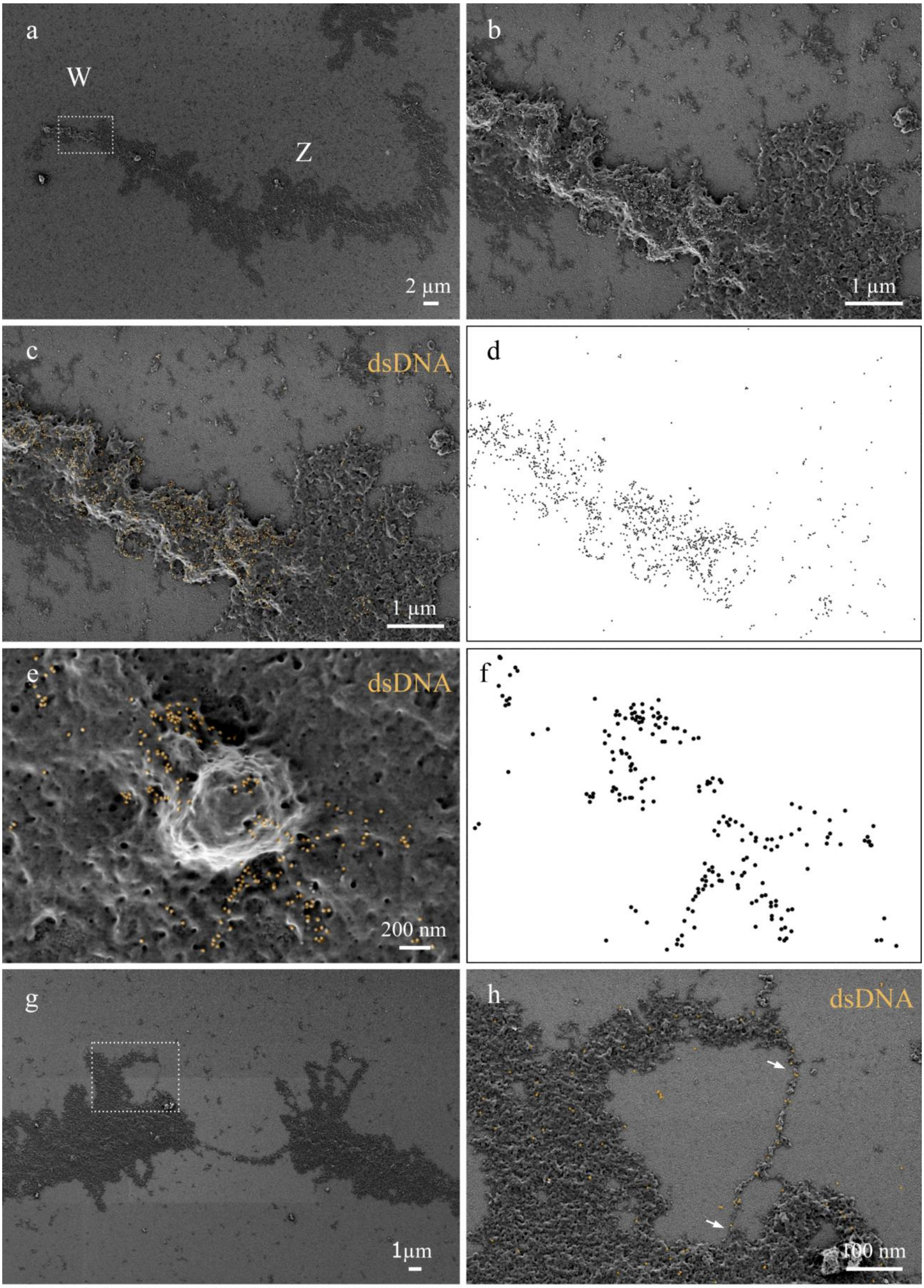
a
W
Z
2 μm
b
1 μm
c
dsDNA
1 μm
d
e
dsDNA
200 nm
f
g
1μm
h
dsDNA
100 nm

Supplementary Figure 2. Surface distribution of dsDNA on chromomeres and untranscribed regions of lateral loop axes. Lampbrush chromosomes of *Coturnix coturnix japonica*, immunogold labelling with antibodies against dsDNA. ZW-lampbrush bivalent (**a**) and enlarged fragment of W-lampbrush chromosome (**b, c, d**), a separate chromomere (**e**), fragment of a lampbrush chromosome (**g**), enlarged lateral loop; edges of untranscribed region marked with arrows (**h**). **b** – dsDNA revealed by 18 nm gold nanoparticles (electron dense granules); **c, e, h** – gold nanoparicles pseudocolured with yellow; **d, f** – distribution pattern of gold nanoparticles. Low-voltage scanning electron microscopy. Scale bars: **a** – 2 μm, **b** – 1 μm, **c** – 1 μm **e** – 200 nm, **g**, **h** – 1 μm, **h**.

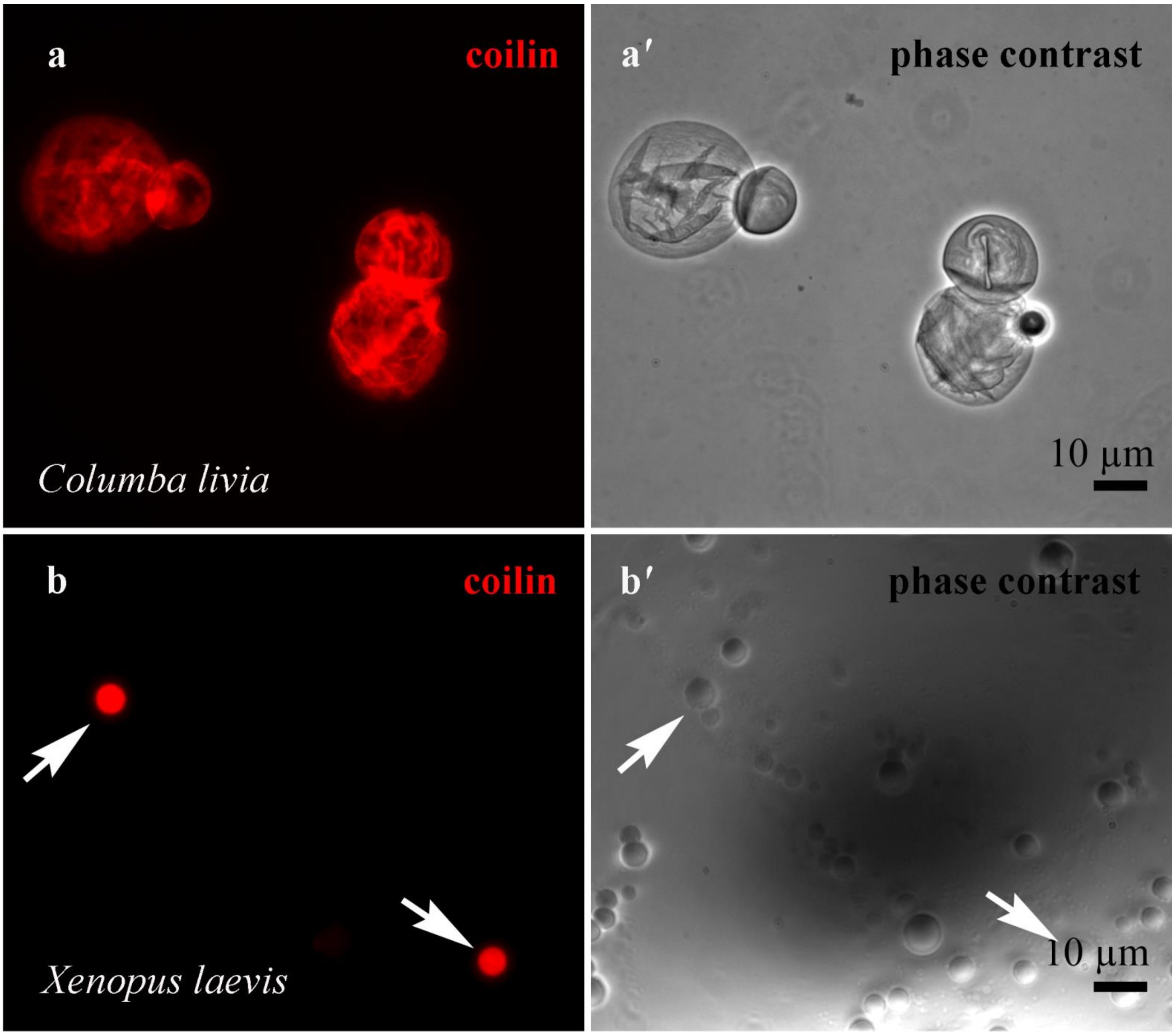

Supplementary Figure 3. Coilin-containing nuclear bodies in *Columba livia* and *Xenopus laevis* growing oocyte nucleus. Distribution of protein coilin in nuclear structures of pigeon (**a**) and frog (**b**) growing oocytes. Immunofluorescent labeling with antibodies against coilin (red). Corresponding phase contrast images in transmitted light are shown (**a′, b′**). Scale bars: 10 μm.

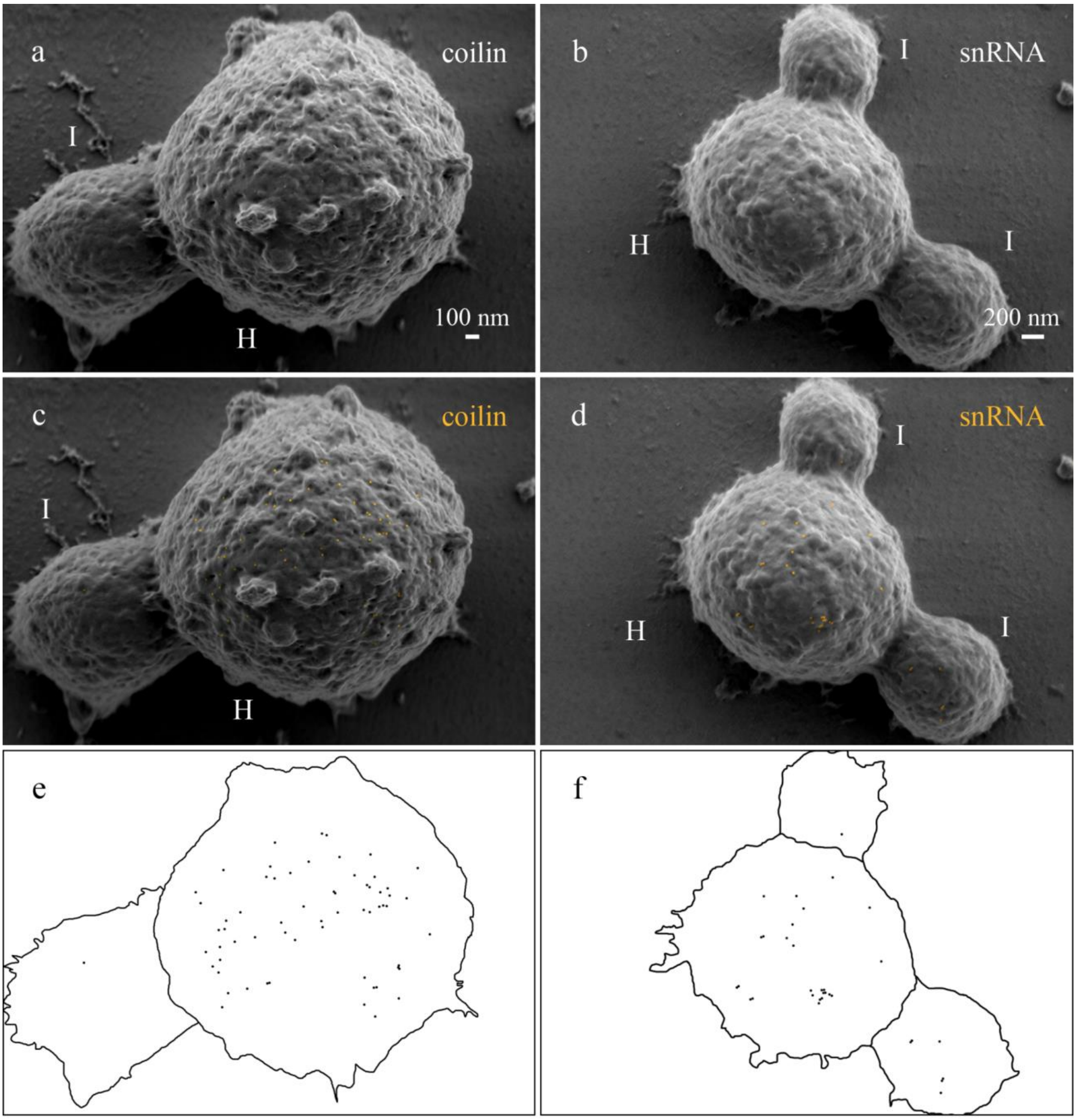

Supplementary Figure 4. The distribution of coilin and snRNA on the surface of HLB and attached IGC of *Xenopus laevis* growing oocytes. Immunogold labeling with antibodies against coilin (**a, c, e**) and snRNAs (**b, d, f**). **a, b** – distribution of coilin or snRNA on the surface of HLB (H) and IGC (I) revealed by 10 nm and 18 nm gold nanoparticles (electron dense granules) correspondingly; **c, d** – gold nanoparicles pseudocolured with yellow; **e, f** – distribution patterns of gold nanoparticles. Low-voltage scanning electron microscopy. Scale bars: **a** – 100 nm, **b** – 200 nm.
